# Quality factor control of mechanical resonators using variable phononic bandgap on periodic microstructures

**DOI:** 10.1038/s41598-021-04459-2

**Published:** 2022-01-10

**Authors:** Naoki Inomata, Yuka Tonsho, Takahito Ono

**Affiliations:** Graduate School of Engineering, Tohoku University, Sendai, 980-8579 Japan

**Keywords:** Electrical and electronic engineering, Mechanical engineering

## Abstract

The quality factor (*Q-*factor) is an important parameter for mechanical resonant sensors, and the optimal values depend on its application. Therefore, *Q-*factor control is essential for microelectromechanical systems (MEMS). Conventional methods have some restrictions, such as additional and complicated equipment or nanoscale dimensions; thus, structural methods are one of the reasonable solutions for simplifying the system. In this study, we demonstrate *Q-*factor control using a variable phononic bandgap by changing the length of the periodic microstructure. For this, silicon microstructure is used because it has both periodicity and a spring structure. The bandgap change is experimentally confirmed by measuring the *Q-*factors of mechanical resonators with different resonant frequencies. The bandgap range varies depending on the extended structure length, followed by a change in the *Q-*factor value. In addition, the effects of the periodic structure on the *Q-*factor enhancement and the influence of stress on the structural length were evaluated. Although microstructures can improve the *Q-*factors irrespective of periodicity; the result of the periodic microstructure is found to be efficient. The proposed method is feasible as the novel *Q-*factor control technique has good compatibility with conventional MEMS.

## Introduction

Mechanical resonators are potent tools for measuring various physical parameters in the microelectromechanical system (MEMS) field, especially for highly sensitive physical sensors^1–5^. The quality factor (*Q-*factor) and its control techniques are one of the key parameters for mechanical resonant sensors because the optimal values depend on its applications, i.e., high resolution and low response speed resulting from a higher *Q-*factor and vice versa. Electrical feedback methods^6–10^, optical pumping^11–19^, mechanical pumping^20–24^, thermal pumping^25–28^, and parametric pumping^29–34^ are conventionally used for *Q-*factor control. Nonlinear damping is another method that can be made effective by changing the vibration amplitude of the nanomechanical resonators^35^. The detailed information regarding the method is mentioned in Ref.^36^. However, these methods have some restrictions, such as additional electrical equipment or nanoscale dimensions. As the phononic bandgap has a strong relationship with the *Q-*factor, a previous study used a phononic pattern to improve the *Q-*factor of a mechanical resonator^37^. The pattern that was fabricated near the support part of the resonator prevented energy loss. Then, *Q-*factor was improved to 10,448 from 2376 with and without the pattern, respectively.

**Figure 1:**
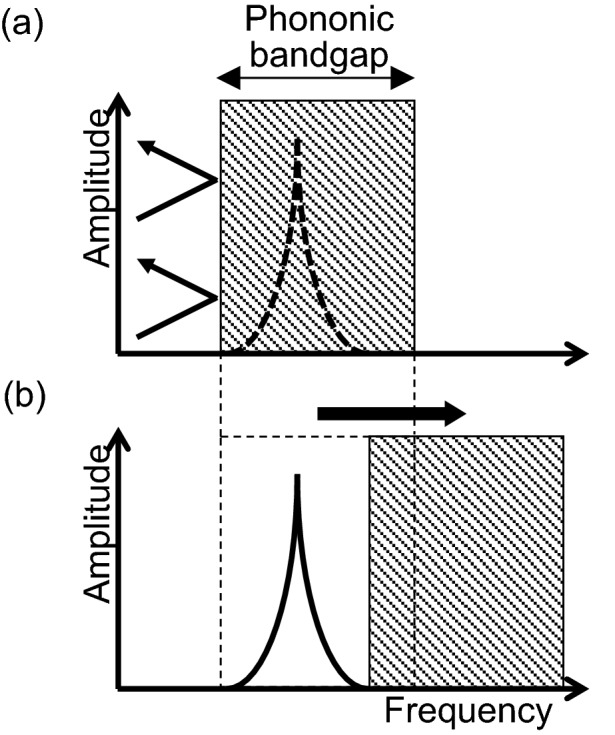
Concept of *Q-*factor control using a variable phononic bandgap. (**a**) A *Q*-factor is “not” observed because of rejecting external vibrations in the case of the resonant frequency is “inside” the phononic bandgap. (**b**) The *Q*-factor is observed because of accepting external vibrations in the case of the resonant frequency is “outside” the phononic bandgap. [Drawn with Microsoft^®^ PowerPoint^®^ for Microsoft 365 MSO, ver. 2110 (https://www.microsoft.com/en-us/microsoft-365/microsoft-office)].

Here, we propose the *Q-*factor can be controlled by changing the phononic bandgap range as shown in Fig. 1. Methods to deform micro-objects by applying force are well established in the MEMS field (e.g., electrostatic or piezoelectric actuators). Therefore, when the periodic microstructure around the mechanical resonator is deformed, the bandgap range changes. Although the resonant frequency can control the *Q*-factor in/out of the bandgap range, there are no such reports.

Several theoretical and experimental reports are available on the variable phononic bandgap in large-scale objects. Robillard et al. theoretically analyzed the change in the phononic bandgap using the strain of magnetostrictive materials^38^. The study was conducted with an assumption of 1 mm squares periodic structures with a period of 1.35 mm and reported an estimated change of 0.5–1.04 MHz bandgap range. Croënne et al. reported a phononic structure by laminating a piezoelectric ceramic and a polymer to form piezocomposites. The size of the composite material was 10 mm square and 1.6 mm thick for one unit, and it was confirmed that the bandgap varied near 1 MHz theoretically and experimentally. The experiment results presented a bandgap change with respect to the applied electrical conditions^39^. Shim et al. theoretically analyzed the change in bandgap by extending the length of a periodic hole structure made of an elastic polymer^40^. The study demonstrated the relationship between the hole radius and the structural period of the pattern. Although the results did not describe a specific frequency range, the bandgap was changed by mechanically changing the structure.

As mentioned earlier, even though there are no reports, it is still realized that the *Q-*factor can be controlled at a high probability using the variable phononic bandgap, especially for silicon (Si) periodic microstructures, which is used widely in MEMS. In this study, we evaluated the *Q-*factor control of mechanical resonators by changing the Si microstructure’s period and pattern. In addition, it was assumed that the *Q-*factor was influenced by changing the structure length because mechanical stress was applied to the microstructure, and we evaluated the influence of stress on the *Q-*factor by mechanical extension.

## Design and fabrication

**Figure 2:**
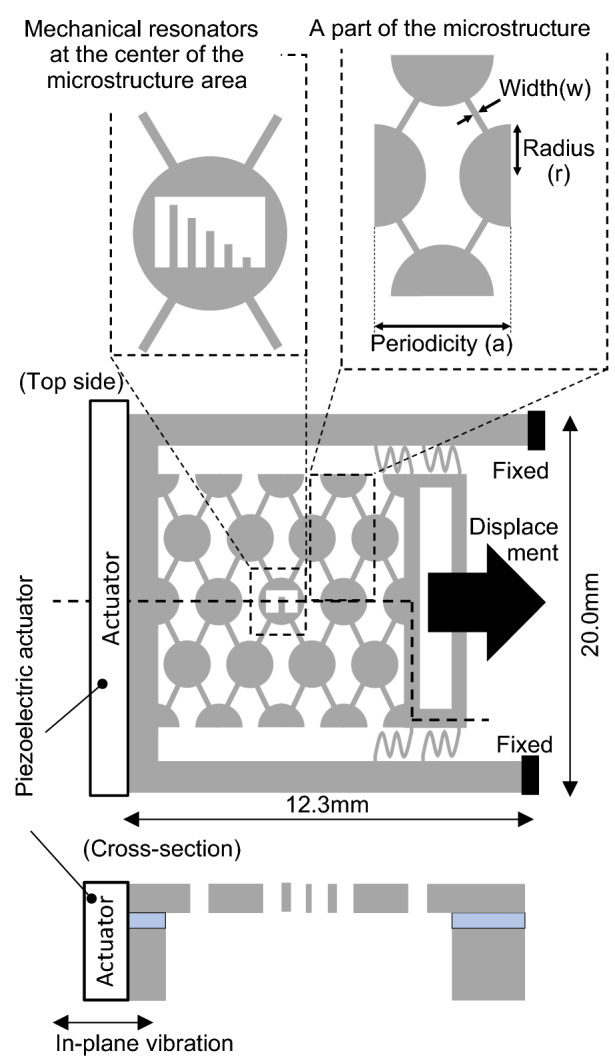
Concept of the proposed device with periodic microstructure. Mechanical resonators were at the center area of the microstructure. The periodicity of the microstructure was changed by pulling via a micromanipulator. [Drawn with Microsoft^®^ PowerPoint^®^ for Microsoft 365 MSO, ver. 2110 (https://www.microsoft.com/en-us/microsoft-365/microsoft-office)].

The schematics of the phononic structure device are shown in Fig. 2. A periodic microstructure sample with a phononic bandgap of a few MHz order was prepared. The Si microstructure with periodicity and spring structure characteristics was used to increase the structure length, and this resulted in the change of structure period and phononic bandgap. A rhombus-shaped device was designed with Si discs connected diagonally opposite to each other, and it contributed to the deformation in one direction. The two opposing sides of the periodic microstructure were free and movable in one axis direction, and the structure was pulled using a micromanipulator. An outer frame (3.5 mm × 7.8 mm) and inner frame (1.5 mm × 5.8 mm) were constructed on the side of the structure to which the micromanipulator’s tip was connected. The device was stable, since it was fixed on both sides, and the microstructure moved only when pulled by the manipulator. Furthermore, to support the microstructure area, four springs were constructed on the frame to connect the manipulator. One side of the device was perpendicularly fixed onto a piezoelectric actuator placed in a vacuum chamber. The vibration direction of the actuator corresponded to the direction of the mechanical resonator’s in-plane vibration. A holding pin at the tip of the 1-axis micromanipulator (mechanically connected with the opposite side) was inserted into the holes of the device.

### Theoretical analysis of the microstructure

**Figure 3:**
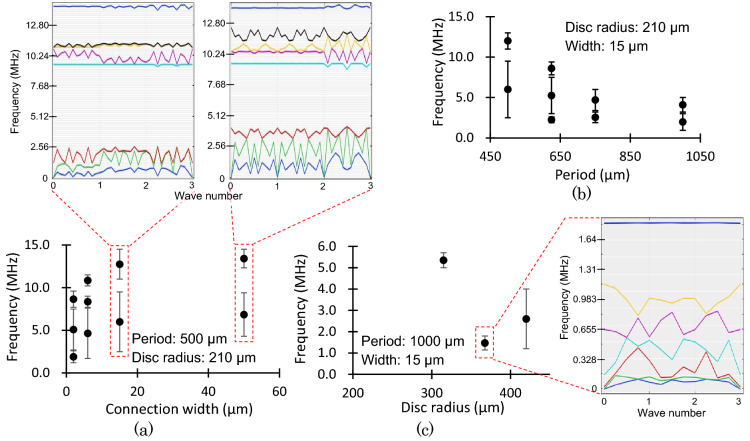
Changes of the phononic bandgap range estimated using FEM analysis and some examples of the phononic band for different (**a**) connection width at constant period and disc radii, (**b**) structure period at constant disc radius and connection width, and (**c**) disc radius at constant period and connection width. The black circle, upper bar, and lower bar depict the bandgap’s median, maximum, and minimum values.

To determine the bandgap range, the relationship between the parameters (the periodic length, disc size (as mass), and connection width) and the bandgap was investigated using the finite element method (FEM) (COMSOL Multiphysics, 2-dimensional solid mechanics interface). In this study, eight frequency solutions were used. In Fig. 3, the black circle, upper bar, and lower bar depict the bandgap’s median, maximum, and minimum values, respectively. Under a constant period and disc radius, the highest and lowest frequencies of the bandgap decreased with a reduction in the connection width (Fig. 3a). As mentioned in Fig. 3b, the bandgap frequency shifted to a lower value for a long structure period. Based on the results from the performed analysis, the connection width between the disc connection and the structure period was 15 µm and 1000 µm, respectively. The structure period corresponded to the length of the connection part between the discs. In our experience, too large width to length structural ratio of the connection part causes low yield for the fabricated samples. Previous studies have determined that phononic bandgap structures have low transmission ratio of < 0.05 in six periods^41^; therefore, our device was designed to have a total of six periods.

Subsequently, the disc radius was determined, and the bandgap was calculated for 200, 315, 367.5, and 420 µm radii. While no bandgap had appeared at 200 µm, a low frequency range was obtained at 367.5 µm (Fig. 3c). The bandgap was dependent on the ratio of structure period to disc radius (a/r). At r/a < 0.3, there were no bandgaps; at r/a > 0.5, the discs were in contact with each other, and the structure could not be established. Furthermore, we decided the disc radius to be 367.5 µm and constructed the mechanical resonators at the center of the microstructure area with different resonant frequencies. The *Q-*factors of these resonators were used to evaluate the phononic bandgap.

**Figure 4:**
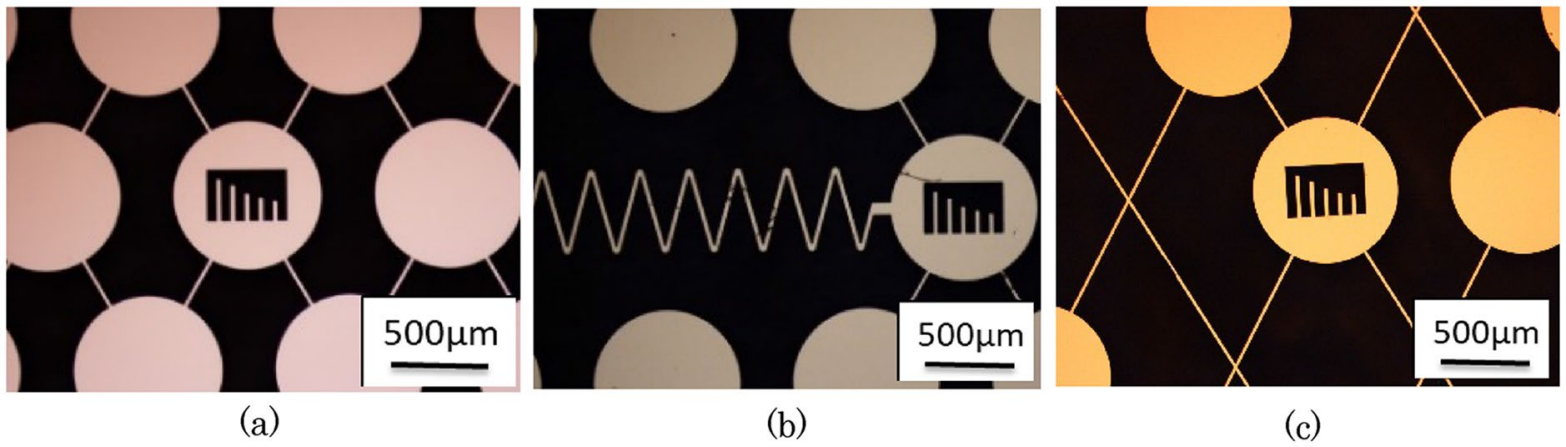
Mechanical resonators and fabricated periodic microstructure (**a**) without waveguide, (**b**) with waveguide, and (**c**) non-periodic microstructure randomly lacking the discs.

The resonant peak of the resonator’s vibration spectrum has a frequency range of approximately 1 kHz (the vibration spectrum can be found in a later “*Q*-factor changes in the phononic bandgap shift” section), and the bandgap required a frequency change larger than the range of 1 kHz. As shown in Fig. 3b, the minimum frequency of the bandgap shifts to 77 kHz when the structure period 1000 µm is extended to 1050 µm. In the constructed design, this 50 µm change per period was possibly extended using a micromanipulator. In the center of the periodic microstructure, mechanical resonators with a frequency of 0.5–6 MHz were assembled. The fabricated structures and resonators are shown in Fig. 4. The devices were fabricated from silicon on insulator (SOI) wafers using conventional microfabrication techniques such as photolithography, Si deep reactive ion etching, and SiO_2_ wet etching. The thickness of the SOI wafer was 56.5 µm, which corresponded to the thickness of the microstructure and width of the in-plane vibrated resonators.

## Experiments and results

**Figure 5:**
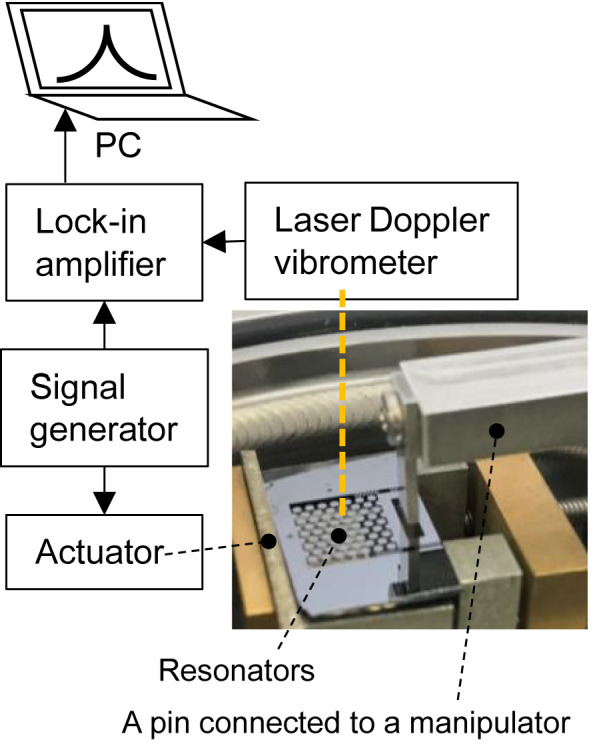
Measurement setup.

The devices in the experimental setup were prepared with periodic microstructure (*N* = 5), periodic microstructure with waveguide to propagate the external vibration (*N* = 3), non-periodic microstructure with randomly lacking discs (*N* = 4), and conventional sample without any microstructure (plain sample) (*N* = 3). *N* indicates the number of studied samples in each category. One side of the device was perpendicularly fixed onto a piezoelectric actuator placed in a vacuum chamber. The vibration spectra of the resonators placed at the center of the microstructure were measured using a laser Doppler vibrometer for in-plane mode (MLD-103A, Neoark Corp.) and a lock-in amplifier (HF2LI, Zurich Instruments) as shown in Fig. 5. We experimentally evaluated the phononic bandgap range by comparing the vibration spectra of each sample, analyzing the effectiveness of the periodic microstructure for *Q*-factor enhancement, and calculating the *Q-*factor change with respect to the microstructure deformation. The vacuum level at the measurements was on the order of 10^–3^ Pa.

### Evaluation of the phononic bandgap

The vibration spectra of the mechanical resonators assembled at the center of the periodic microstructure were measured. For the resonant frequencies of 0.602, 0.832, 1.40, 2.45, 3.70, 5.32, and 5.79 MHz, the vibration could not be observed, whereas it was detected for the rest (Table 1). By contrast, in the non-vibration frequencies of microstructure without waveguide, the vibration spectra were observed for the microstructure with the waveguide. It is determined that the frequency range at which vibration spectra were not observed corresponds to the phononic bandgap. As shown in Table 1, after excluding several frequencies, the bandgap range of the periodic microstructure was 0.6–5.79 MHz.

**Table 1:** Mechanical resonator with (〇) and without (×) vibrations for different resonant frequencies.

	Resonant frequency (MHz)											
	0.602	0.832	1.40	1.98	2.45	2.99	3.43	3.70	4.78	4.85	5.32	5.79
**Displacement (µm)**												
0	×	×	×	〇	×	〇	〇	×	〇	〇	×	×
150	×	×	×	〇	×	〇	〇	×	〇	〇	〇	〇
200	〇	〇	×	〇	×	〇	〇	×	〇	〇	〇	〇
250	〇	〇	×	〇	×	〇	〇	×	〇	〇	〇	〇

### Dependence of *Q-*factor on the microstructure patterns

**Figure 6:**
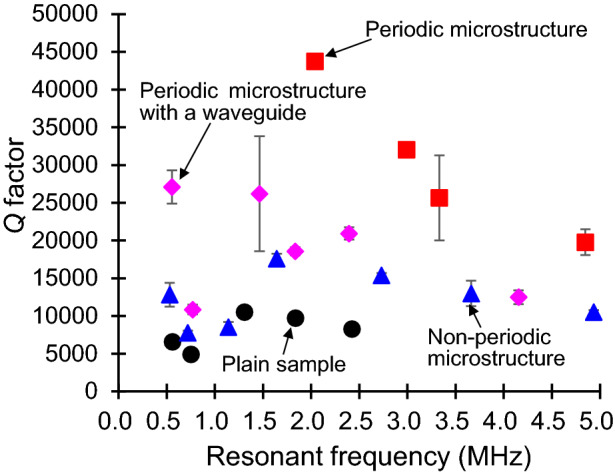
*Q-*factors of the mechanical resonators for different microstructures.

The study compared the *Q-*factors of the periodic microstructure, periodic microstructure with waveguide, non-periodic microstructure, and plain sample. The *Q-*factors were observed to be higher according to the order of periodic microstructure > periodic microstructure with waveguide > non-periodic microstructure > plain sample (Fig. 6). As proposed initially, the periodic microstructure had the highest *Q*-factor.

### *Q*-factor changes in the phononic bandgap shift

**Figure 7:**
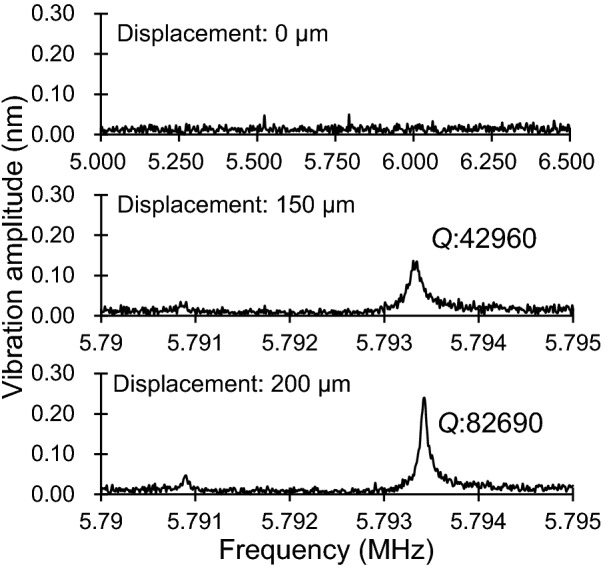
Vibration spectra of the mechanical resonators which has a resonant frequency near the bandgap boundary.

The length of the periodic microstructure was increased by 50 µm using a micromanipulator, and the *Q*-factors of the mechanical resonators were measured. The vibration spectra without displacement were not observed for the resonant frequencies near the bandgap boundary (0.602, 0.832, 5.32, and 5.79 MHz); however, it was observed at larger displacements. For example, the vibration spectra of the resonator with 5.79 MHz resonant frequency are shown in Fig. 7. The vibration spectra with a *Q*-factor of 42,960 and 82,690 appeared at a displacement of 150 µm and 200 µm, respectively.

**Figure 8:**
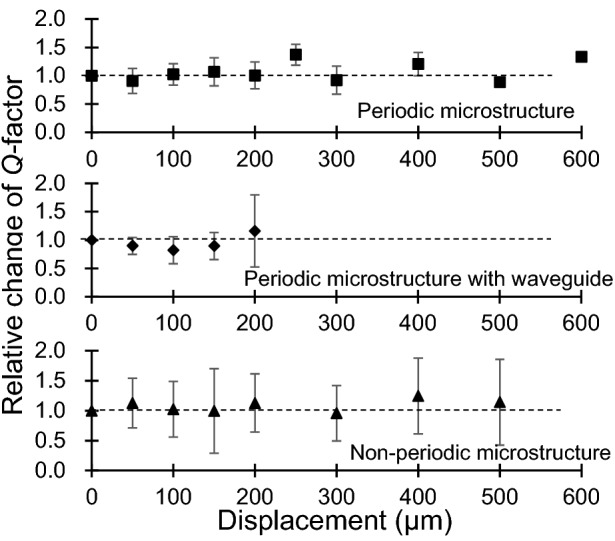
*Q-*factors with respect to displacements for different microstructures.

In addition, the *Q-*factors at each displacement were compared using a resonator whose resonant frequency was out of the phononic bandgap’s range. The influence of the stress that extended the microstructure was evaluated, and the structures were extended until they reached the breaking point. As shown in Fig. 8, no noticeable change in the *Q-*factor was observed for any structure.

## Discussions

The resonant frequencies for which the vibration spectra were not observed in the periodic microstructure were observed for the device with the waveguide. These results indicated that the periodic microstructure had a phononic bandgap, and the external vibrations added to the structure were rejected. For the waveguide scenario, the external vibrations were propagated to the resonator placed at the center of the periodic microstructure. This resulted in the vibration of the resonators that did not vibrate in the periodic microstructure, therefore proving the existence of the phononic bandgap.

**Figure 9:**
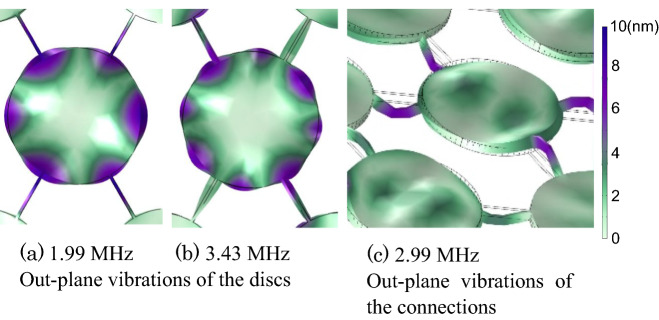
Mechanical vibrations obtained by FEM analysis. Purple and green colors depict large and low deformations. [Drawn with COMSOL Multiphysics, ver. 5.6 (https://www.comsol.com/)].

In the experimentally observed phononic bandgap, discontinuous frequency ranges were measured near 2 MHz and 3.0–3.43 MHz range. We considered the in/out-of-plane mechanical vibration of the structure as an unexpected result. In the FEM analysis of the periodic microstructure vibration, the out-plane vibrations of the discs and in-plane vibration of the connection appeared at 2.0, 3.0, and 3.4 MHz resonant frequencies (Fig. 9). This value was compared to the discontinuous frequency range in the experimental bandgap, and it indicated that the mechanical vibration of the microstructure itself had an influence. The frequency range of the phononic bandgap decreased across the board, extending the length of the periodic microstructure (Table 1). Theoretically, it was clarified that the bandgap frequency was inversely proportional to the length of the periodic microstructure; it decreased for longer periods of the microstructure. Therefore, the theoretical solution corresponds to the experimentally derived results.

The *Q*-factors were observed to be high in the order of periodic microstructure > periodic microstructure with waveguide > non-periodic microstructure > plain sample. In the periodic microstructure with the waveguide, the external vibrations were propagated to the mechanical resonators; however, there were simultaneous energy dispersions via the waveguide. Therefore, the *Q-*factors of the microstructure with the waveguide were low when compared with those without the waveguide. Furthermore, the *Q-*factors of the non-periodic microstructure were higher than those of the plain sample. The energy loss decreased irrespective of the microstructure periodicity because the area of the supporting parts around the resonators were reduced. Although the periodic microstructure was efficient, only the parts surrounding the microstructure had good effects on improving the *Q-*factor.

In addition, as resonant frequency increased, the *Q-*factor decreased, and shorter mechanical resonators had a higher resonant frequency. The *Q-*factor deceased for a larger surface-to-volume ratio (the energy loss from surface increases) and smaller length-to-width ratio (the energy loss via supporting part increases^42,43^. Figure 6 corresponds to the experimental results. Furthermore, there are other factor that influence *Q*-factor such as surface pollution, temperature annealing, and plasma damage. In this study, all samples were fabricated with similar processes and machines; then, it was determined that the influence of these factors on the *Q*-factor did not show significant difference on the measurements.

Some mechanical resonators did not exhibit the vibration spectra in the original periodic length. However, the spectra appeared as the periodic length was increased, which also increased the *Q-*factors. These results indicate that the resonant frequencies of the resonators were within the phononic bandgap range; therefore, the resonators did not vibrate because the external vibration signal was filtered. Gradually, the resonant frequency shifted from the inside to the outside of the bandgap, thereby extending the periodic length, and finally, the resonant frequency exited from the bandgap. Additionally, the boundary of the bandgap was assumed to be gradual and not perpendicular.

The effect of the stress when extending the microstructure length could be evaluated via the *Q-*factors of the resonators whose resonant frequencies were always outside the bandgap. No notable changes in the *Q-*factors were observed for the extended periodic length of all the microstructures (Fig. 8). Therefore, the effect of the stress was limited.

## Conclusion

In this study, we achieved *Q-*factor control of mechanical resonators by changing the phononic bandgap.

The phononic bandgap of the fabricated periodic microstructure was experimentally verified based on the vibration spectra of mechanical resonators. The *Q-*factors changed depending on the phononic bandgap shift with respect to the periodic length of the microstructure. However, the stress that extended the microstructure did not significantly affect the *Q-*factor. In conclusion, we demonstrated the feasibility of controlling the *Q-*factors using phononic bandgap shifts of periodic microstructures, along with on/off vibrations. This technique is compatible with conventional microfabrication processes and well-established actuation methods causing displacement in MEMS and leads a powerful solution to be realized a simple system without complicated external equipment against sensing performance requiring different *Q*-factor depending on situations that switch high sensitivity or high response via mechanical resonators to measure physical parameters.
